# Supplementary material for: Causal Relationship between Aging and Anorexia Nervosa: A White-Matter-Microstructure-Mediated Mendelian Randomization Analysis
Source: Biomedicines. 2024 Aug 16;12(8):1874. doi: 10.3390/biomedicines12081874 (PMC11351342; doi:10.3390/biomedicines12081874)
Supplement: Supplementary file 1 [file biomedicines-12-01874-s001.zip › Supplementary Figure/Figure S4/Singe SNP plot of MR analysis for TL to ubm-b-1819.pdf]

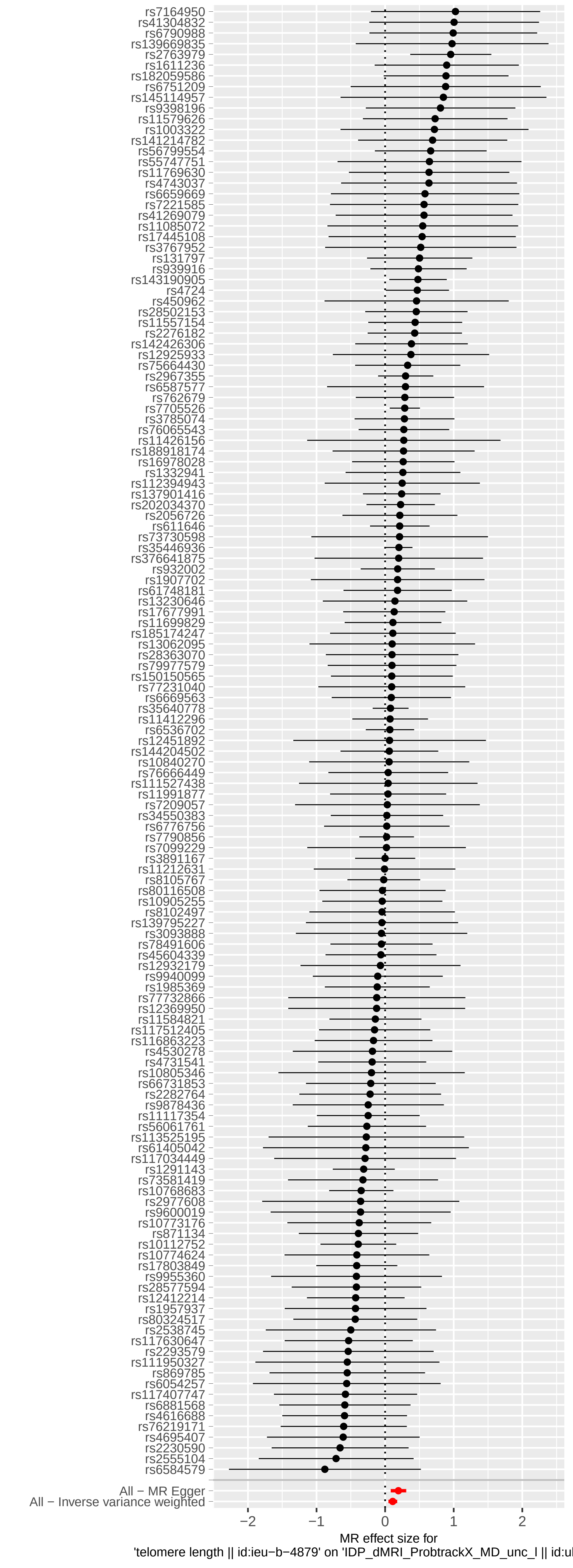
rs7164950
rs41304832
rs6790988
rs139669835
rs2763979
rs1611236
rs182059586
rs6751209
rs145114957
rs9398196
rs11579626
rs1003322
rs141214782
rs56799554
rs55747751
rs11769630
rs4743037
rs6659669
rs7221585
rs41269079
rs11085072
rs17445108
rs3767952
rs131797
rs939916
rs143190905
rs4724
rs450962
rs28502153
rs11557154
rs2276182
rs142426306
rs12925933
rs75664430
rs2967355
rs6587577
rs762679
rs7705526
rs3785074
rs76065543
rs11426156
rs188918174
rs16978028
rs1332941
rs112394943
rs137901416
rs202034370
rs2056726
rs611646
rs73730598
rs35446936
rs376641875
rs932002
rs1907702
rs61748181
rs13230646
rs17677991
rs11699829
rs185174247
rs13062095
rs28363070
rs79977579
rs150150565
rs77231040
rs6669563
rs35640778
rs11412296
rs6536702
rs12451892
rs144204502
rs10840270
rs76666449
rs111527438
rs11991877
rs7209057
rs34550383
rs6776756
rs7790856
rs7099229
rs3891167
rs11212631
rs8105767
rs80116508
rs10905255
rs8102497
rs139795227
rs3093888
rs78491606
rs45604339
rs12932179
rs9940099
rs1985369
rs77732866
rs12369950
rs11584821
rs117512405
rs116863223
rs4530278
rs4731541
rs10805346
rs66731853
rs2282764
rs9878436
rs11117354
rs56061761
rs113525195
rs61405042
rs117034449
rs1291143
rs73581419
rs10768683
rs2977608
rs9600019
rs10773176
rs871134
rs10112752
rs10774624
rs17803849
rs9955360
rs28577594
rs12412214
rs1957937
rs80324517
rs2538745
rs117630647
rs2293579
rs111950327
rs869785
rs6054257
rs117407747
rs6881568
rs4616688
rs76219171
rs4695407
rs2230590
rs2555104
rs6584579
All − MR Egger
All − Inverse variance weighted
−2
−1
0
1
2
MR effect size for
'telomere length || id:ieu−b−4879' on 'IDP_dMRI_ProbtrackX_MD_unc_l || id:ul
